# Habitat and Haplotype‐Specific Genetic Vulnerability Analysis Combined With a Multidimensional Scoring System Provides a New Insight for Conservation Prioritization of *Ephedra przewalskii*

**DOI:** 10.1002/ece3.72614

**Published:** 2025-12-14

**Authors:** Hongchao Wang, Qi'ao Ma, Liwei Wang, Ying Li, Meng Cheng, Xiaolin Li, Guang Yang, Kai Sun, Xiulian Chi

**Affiliations:** ^1^ State Key Laboratory for Quality Ensurance and Sustainable Use of Dao‐di Herbs, National Resource Center for Chinese Materia Medica, China Academy of Chinese Medical Sciences Beijing China

**Keywords:** anthropogenic activities, climate change, climate‐niche factor analysis, ensemble modeling approach, *Ephedra przewalskii*, haplotype

## Abstract

*Ephedra przewalskii* is a key species in arid regions recognized for its remarkable ecological, economic, and medicinal importance. However, climate change and anthropogenic activities have severely threatened their survival, reduced genetic diversity, and increased the risk of extinction. Nonclimatic variables lack systematic integration in current modeling frameworks, and intraspecific genetic variation is similarly poorly explored. This study assessed the vulnerability and adaptive capacity of *E. przewalskii* populations by applying ensemble species distribution models (ESDMs) and Climate‐Niche Factor Analysis (CNFA), incorporating climate scenarios and human activity data. Additionally, we calculated the haplotype genetic vulnerability and constructed a multidimensional scoring system that combined habitat and genetic vulnerabilities. High vulnerability was observed in regions such as Nilka County and Hotan, whereas regions such as the Ejina Banner‐Hami‐Heiying Mountain‐Jiuquan range area exhibited reduced vulnerability for *E. przewalskii* . The habitat vulnerability decreased over time under Shared Socioeconomic Pathways 1‐2.6 (SSP1‐2.6) but significantly increased by the 2090s under Shared Socioeconomic Pathways 5‐8.5 (SSP5‐8.5). Haplotypes G, E, and A were identified as being at high risk for genetic diversity loss under SSP5‐8.5. Application of the multidimensional scoring system successfully identified and prioritized key conservation hotspots—including populations in Wuheshalu, Qiemo, and Ruoqian (Tarim Basin) as well as Karamay, Wuchang, and Burqin (Junggar Basin)—based on their high haplotype diversity and vulnerability. The approach thereby offers an adaptable framework for vulnerability assessment and supporting broader conservation efforts.

## Graphical abstract

This study assesses the vulnerability and adaptive capacity of *Ephedra przewalskii* populations using ensemble species distribution models and Climate‐Niche Factor Analysis, integrating climate and human activity data. It reveals that habitat fragmentation and shrinkage are exacerbated by climate change and anthropogenic activities, with varying genetic vulnerability among haplotype populations. A newly constructed multidimensional scoring system helps prioritize conservation efforts, offering crucial insights for the species' conservation and a general framework for assessing other species' vulnerability.

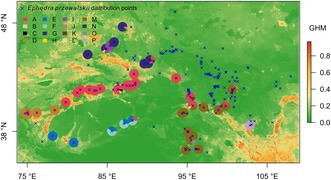

## 1 Introduction

*Ephedra przewalskii* Stapf is an extremely xerophytic species native to northwestern China and Mongolia and is included in the International Standard Appendix for the genus *Ephedra* by the International Organization for Standardization (ISO) (Wu and Hong 1999; Wu et al. 2010; Su and Zhang 2016; ISO 2023). This perennial shrub thrives in hyper‐arid, soil‐degraded environments such as the Gobi Desert and alluvial plains in the foothills (Wu and Hong 1999; Pan et al. 2003; Z. Wang 2024). As a desert indicator species (Su and Zhang 2016), *E. przewalskii* demonstrates notable resilience and can coexist with or without other drought‐resistant plants, forming robust, drought‐resistant ecosystems that are critical for desert land conservation and ecological restoration (Liu et al. 2018). In addition, *E. przewalskii* has considerable economic potential, serving as a high‐quality fuelwood and an important source of traditional Chinese medicinal materials (Xie et al. 2010; Huang and Li 2017). The stems exhibit analgesic, anticancer, and anti‐influenza activities, making them effective clinical alternatives to the stems of other *Ephedra* species (ISO 2023). Notably, its extract exhibits stronger antiviral activity against SARS‐CoV‐2 than that of the pharmacopeia‐documented extract of *Ephedra sinica* Stapf, indicating that it is a promising alternative for treating various viral infections (Kakimoto et al. 2024). Its morphological similarity to the medicinal *Ephedra intermedia* leads to frequent misidentification in trade, which contributes to considerable population declines and threatens wild resource conservation (Ma et al. 2018). Furthermore, climate oscillations influence *E. przewalskii* 's evolutionary processes (allopatric divergence and range shifts in population distribution) (Su and Zhang 2016). The combined pressures of persistent anthropogenic activities and climate change now pose substantial threats to this species, potentially exacerbating range contraction, habitat fragmentation, and genetic divergence despite its inherent adaptive capacity (Su and Zhang 2016). However, quantitative assessments of the environmental risks and adaptive capacities remain limited.

Climate change and anthropogenic pressure are among the most urgent global threats to biodiversity (Shrestha et al. 2021). Critically, climate change impact assessments that exclude land‐use changes may mischaracterize the vulnerability and spatiotemporal distribution of medicinal plants (Feng et al. 2025). Their synergistic effects, including habitat destruction, species introduction, resource exploitation, industrial pollution, and increased CO_2_ concentrations, profoundly disrupt interspecies interactions, resulting in community reorganization and potential species extinction (Parmesan and Yohe 2003; Root et al. 2003; Urban 2015; Daru et al. 2017; Theobald et al. 2020; Shrestha et al. 2021; Storch et al. 2021; Wang et al. 2022; Soga and Gaston 2024). These impacts could lead to the extinction of approximately 1 million plant and animal species (Barnosky et al. 2011; Dobson et al. 2021; Song et al. 2021). The dryland ecosystems, which cover 41% of the Earth's land surface, support unique flora, sustain human livelihoods, and are particularly vulnerable (Safriel and Adeel 2008; Chen et al. 2022; Zhang et al. 2023). However, our understanding of the specific effects of climate change and anthropogenic activities on plant distribution in arid regions remains limited (Sun et al. 2021; Zhang, Chen, et al. 2024).

Although an increasing body of research has explored global change and anthropogenic pressures on community‐level species diversity, considerably less focus has been directed toward intraspecies genetic variation (Pauls et al. 2013; Pelletier and Coltman 2018; Caldwell et al. 2024). Intraspecific genetic diversity, which typically varies across intraspecific genetic groups, is a key focus of conservation biology, particularly for vulnerable species (Jing et al. 2023). However, population‐level evolutionary adaptations rarely maintain the same momentum with the rapid climatic or anthropogenic changes (Morgan et al. 2020; Mattila et al. 2024). Because evolution relies on multigenerational trait accumulation and gradual gene frequency shifts, environmental changes that exceed a species' adaptive capacity may result in insufficient adaptive variation within populations, potentially leading to local extinction (Pauls et al. 2013; Alekseeva et al. 2020). However, climate change and anthropogenic activities are primary drivers of genetic divergence (Tilman and Lehman 2001; Su and Zhang 2016). Clarifying their combined effects on intraspecific genetic diversity is critical for guiding conservation and sustainable use; however, the response of distinct genetic populations (influenced by adaptive evolution) to these pressures remains unclear (Diniz‐Filho and Bini 2019; Waldvogel et al. 2020; Sang et al. 2022; Mattila et al. 2024).

Species vulnerability assessments quantify climate‐driven extinction risks and inform conservation strategies (Wang et al. 2022). Since the Intergovernmental Panel on Climate Change (IPCC) formalized its vulnerability framework, methodologies for evaluating distinct vulnerability components have proliferated (Pacifici et al. 2015; Foden et al. 2018; Leclerc et al. 2020). Climate‐Niche Factor Analysis (CNFA) is a robust tool that leverages species occurrence data and bioclimatic variables to assess climate sensitivity and exposure (Rinnan and Lawler 2019; Jamwal et al. 2022; Guo et al. 2024). Genomic advances have improved climate vulnerability assessments of plant populations; however, most studies have focused solely on climatic variables and neglected nonclimatic drivers (Gougherty et al. 2021; Sang et al. 2022; Hou et al. 2024; Yang et al. 2024). Although single‐nucleotide polymorphisms (SNPs) provide insights into genetic variation, haplotype analysis offers superior resolution for characterizing regional genetic diversity and predicting the risk of genetic loss (Ma et al. 2017; Li et al. 2018; Bhat et al. 2021). The CNFA addresses these limitations through its unique capacity to incorporate nonclimatic variables (Rinnan and Lawler 2019). By quantifying habitat vulnerability under combined climatic and anthropogenic pressures, this approach integrates ecological and genetic assessments and ultimately clarifies the adaptive capacity and evolutionary potential of species in complex environments.

Therefore, we constructed ensemble Species Distribution Models (SDMs) to project and determine the potential habitat of *E. przewalskii* under current environmental conditions. Subsequently, CNFA was applied to evaluate the habitat and haplotype‐specific genetic vulnerability of *E. przewalskii* to climatic variables and anthropogenic pressures. A multidimensional scoring system was developed to identify hotspot populations of *E. przewalskii* . This study aimed to (1) quantify and map future habitat vulnerability patterns under climate change and anthropogenic activities, (2) compare genetic vulnerability differences among haplotypes, and (3) propose targeted conservation strategies for *E. przewalskii* .

## 2 Materials and Methods

### 2.1 Data Acquisition

#### 2.1.1 Geographic Distribution

**FIGURE 1:**
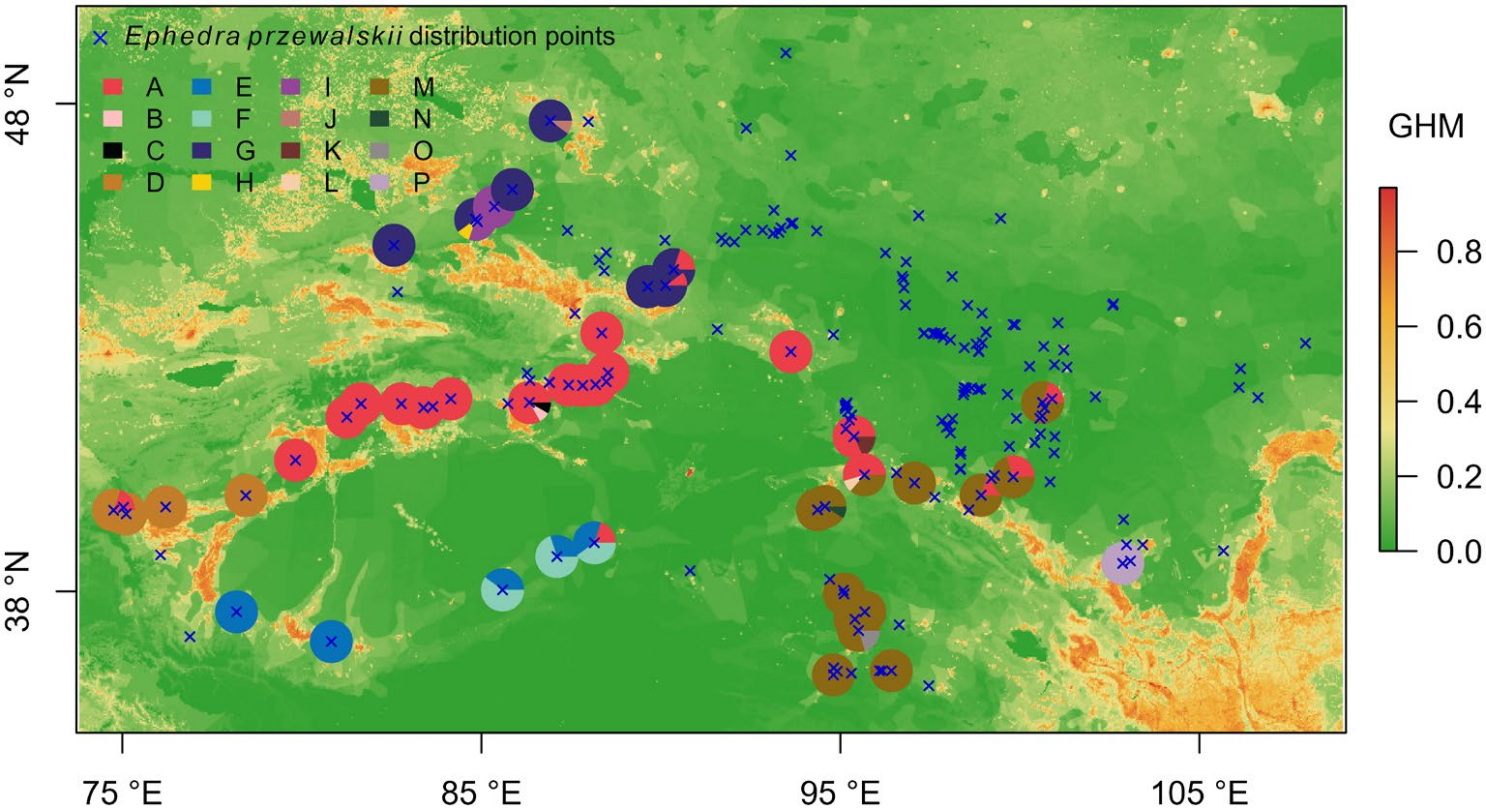
Geographic distribution points of *Ephedra przewalskii* and the proportions of haplotype composition at 45 sampled distribution points. GHM = Global Human Modification (anthropogenic activities index, https://figshare.com/articles/dataset/Global_Human_Modification, accessed on March 12, 2024). Haplotype A–P are 16 haplotypes of *E. przewalskii* identified by Su and Zhang (2016).

A total of 590 wild geographic distribution points of *E. przewalskii* were compiled from multiple sources (Table S1), including fieldwork, literature records (Zhang, Chang, et al. 2015; Zhang, Li, et al. 2015; Su and Zhang 2016; Ma et al. 2018; Li 2020; Alus 2021; Xing and Zhang 2021; Meng et al. 2022; Han et al. 2024; Pironon et al. 2024), the Chinese Virtual Herbarium (CVH, http://www.cvh.org.cn/cms/), Plant Science Data Center of Chinese Academy of Sciences (CAS PSDC, https://www.plantplus.cn/), and Global Biodiversity Information Facility (GBIF, https://www.gbif.org/). These data points were then deduplicated and filtered based on the range of species recorded in *Flora of China* (Wu and Hong 1999) and the aridity index (Zomer et al. 2022). Only records from 1992 onwards were retained to account for the substantial changes in anthropogenic activities over recent decades (Bruckmeier 2024). Additionally, we performed spatial filtering by retaining one distribution point per 1 km × 1 km grid (matching the resolution of the environmental layers) to reduce spatial autocorrelation, which is a key step in reducing overfitting due to sampling bias in ecological niche models (Boria et al. 2014). After this filtering process, 186 distribution points remained for further analysis (Table S1). The study area extended by 1° to the east, west, south, and north, resulting in a final study area spanning 35.1°–50.0° N and 73.8°–109.0° E. The geographic locations of these distribution points and the spatial patterns of anthropogenic activities are shown in Figure 1.

Among these 186 distribution points, 45 were derived from a study by Su and Zhang (2016), who identified 16 haplotypes (A–P) by sequencing chloroplast DNA (cpDNA) fragments from 469 individuals across 45 populations of *E. przewalskii* (Table S2). The 45 sampled distribution points were used for haplotype‐based vulnerability analyses to assess the risk of genetic diversity loss in *E. przewalskii* . This approach provides a robust scientific foundation for the development of genetic diversity conservation strategies for this species.

#### 2.1.2 Climate Data

Current and future climate data were obtained from WorldClim (https://www.worldclim.org; accessed July 18, 2024). For current conditions, global average climate data from WorldClimv2.1, which compiled records for the period 1970–2000, was used (Fick and Hijmans 2017). For future projections, two time periods were considered: 2041–2060 (the 2050s) and 2081–2100 (the 2090s). To account for future climate uncertainty, three global climate models (GCMs) were selected: HadGEM3‐GC31‐LL (UK Met Office Hadley Center), CMCC‐ESM2 (Centro Euro‐Mediterraneo sui Cambiamenti Climatici), and IPSL‐CM6A‐LR (Institut Pierre‐Simon Laplace), along with two Shared Socioeconomic Pathways (SSPs): SSP1‐2.6 and SSP5‐8.5. Comparing these scenarios enabled the exploration of climate change impacts on *E. przewalskii* and the associated shifts in vulnerability. For all the climate scenarios described above, 19 bioclimatic variables (Bio 1–19) at a resolution of 30 arcsec were first downloaded and clipped to the study area. To avoid model overfitting and reduce autocorrelation from highly correlated environmental variables (de Oliveira et al. 2014; Varela et al. 2014), four strategies were used to screen key variables that influence *E. przewalskii* distribution. (1) Logistic regression analysis: 1000 random pseudo‐absence points were merged with 186 presence points. Using R 4.3.1's glm() (base package) and ecospat.adj.D2.glm() (ecospat package) (Di Cola et al. 2017), we calculated the adjusted *D* ^2^ values for 19 climatic variables; higher values indicated stronger effects on distribution (Figure S1a). (2) MaxEnt suitability projection: Bioclimatic variables and species data were imported into MaxEnt v.3.4.1 for suitability projection. Variables contributing more than 0.05% to the model variation were retained (Figure S1a). (3) Correlation analysis: Pearson's correlation coefficients (*r*) were computed for all 19 environmental variables. Only variables with |*r*| < 0.75 were selected to exclude redundancy from highly correlated pairs (|*r*| ≥ 0.75; Figure S1b), as highly correlated predictors typically carry similar spatial gradients that can propagate autocorrelation into model residuals. (4) Literature review: Key variables were identified by reviewing studies on Ephedra's growth environment (Su and Zhang 2016; Liu et al. 2023; B. Wang 2023) to prioritize ecologically relevant factors. Based on these results, five climatic variables were selected for the SDMs and CNFA: Bio 13 (Precipitation of Wettest Month), Bio 9 (Mean Temperature of Driest Quarter), Bio 12 (Annual Precipitation), Bio 8 (Mean Temperature of Wettest Quarter), and Bio 19 (Precipitation of Coldest Quarter).

#### 2.1.3 Human Activity Data

The human activity data were derived from a Global Human Modification (GHM) dataset (Kennedy et al. 2019). This 1 km^2^ resolution dataset integrates 13 global indicators across five categories: (a) human settlements, (b) agriculture, (c) transportation, (d) mining and energy production, and (e) electrical infrastructure. GHM data have been widely utilized in ecosystem research because of their reliability and comprehensive data on contemporary anthropogenic impacts (Theobald et al. 2020; Meng et al. 2023). The downloaded GHM data were clipped to the study area for further analysis.

### 2.2 Habitat Identification

Considering that species distribution models (SDMs) can enhance projection accuracy by incorporating multiple environmental variables and identifying key drivers of species distribution, they are widely used for habitat identification (Williams et al. 2009; Gogol‐Prokurat 2011; Jamwal et al. 2022). The habitat range of *E. przewalskii* was defined based on its current potential distributions projected by ensemble SDMs that integrated the effects of climatic and human activity variables. Initially, six extensively used algorithms were employed to simulate the current distribution: Classification Tree Analysis (CTA), Generalized Linear Models (GLM), Multivariate Adaptive Regression Splines (MARS), Artificial Neural Networks (ANN), Maxent Entropy (MaxEnt), and Random Forest (RF). Owing to the availability of species presence data only, three groups of pseudo‐absence data (500 points each) were randomly generated. Each presence–absence dataset was then split into two subsets for cross‐validation, with 75% allocated to model training and 25% reserved for validation. To mitigate the bias from random selection, the partitioning of the training and evaluation subsets was repeated three times. Ultimately, 54 SDMs were run and calculated as six algorithms × three pseudo‐absence datasets × three cross‐validation repetitions. Second, only SDMs with an AUC > 0.85 were retained for ensemble forecasting of *E. przewalskii* 's current potential distribution. Four ensemble forecasting methods were used to integrate the projected distribution probabilities: committee averaging (EMca), mean (EMmean), median (EMmedian), and weighted mean (EMwmean). Binarization probability thresholds were automatically determined via receiver operating characteristic (ROC) curves, converting ensemble distribution probabilities into binary presence–absence values. A conservative approach, hereafter referred to as the consensus ensemble model (CEM), was further used, and a grid cell was considered to contain the species only if all four ensemble models confirmed their presence. To validate the effectiveness of our strategies for reducing spatial autocorrelation, Moran's *I* statistic was used to evaluate spatial autocorrelation in the residuals of these four ensemble models and the CEM. The CEM showed a nonsignificant residual spatial autocorrelation (Moran's *I*: −0.0040 to −0.0033, *p*_adj_ > 0.05; Table S3). Therefore, the current potential distribution areas of *E. przewalskii* projected by the CEM were identified as habitat ranges. To integrate these projections into the CNFA framework, the raster data of the potential distribution areas were converted into a polygonal vector layer and exported in Shapefile format.

### 2.3 Vulnerability Analyses

CNFA was used to assess *E. przewalskii* 's vulnerability to environmental change using the “CENFA” package (Rinnan and Lawler 2019). CNFA assumes ecological niche stability by analyzing species sensitivity and exposure to environmental changes to synthesize and quantify their vulnerability to future climate change (Rinnan and Lawler 2019). We assumed that the human footprint remained unchanged for all future vulnerability assessments. Additionally, the uncertainty in future climate projections across GCMs can influence exposure assessments and bias species vulnerability evaluations (Wang et al. 2021, 2022). To address this issue, we averaged the vulnerability indices from multiple GCMs to generate a unified vulnerability pattern. The “bias = 2” parameter was applied in vulnerability mapping to adjust color gradient skewness, enhancing the distinguishability of vulnerable areas for easier identification and analysis.

### 2.4 Genetic Diversity Loss Risk Assessment

To assess the risk of genetic diversity loss in *E. przewalskii* owing to climate change and anthropogenic activities, we compared the vulnerability differences among haplotypes across their distribution areas using data from Su and Zhang (2016). They identified 16 haplotypes of *E. przewalskii* across 45 sampling points. From these, we extracted vulnerability values and retained only haplotypes with at least three sampling points to ensure robust analysis. A rarefaction analysis of haplotype richness validated sampling sufficiency for the selected six target haplotypes; the curve plateaued at 19 sampling points, and additional sampling did not substantially increase haplotype counts (Figure S2), ensuring that subsequent analyses based on these haplotypes were not biased by insufficient sampling.

Pre‐analysis of vulnerability data for these six haplotypes indicated that it met the requirements for normality and homoscedasticity (Shapiro–Wilk tests: *p* ∈ [0.085, 0.271]; Bartlett tests, *p* ∈ [0.080, 0.304]). Therefore, analysis of variance (ANOVA) was used to assess significant differences in vulnerability among these haplotypes. Where significant differences were detected, post hoc multiple comparison tests were conducted to elucidate detailed variations in vulnerability characteristics, and adjusted *p* values were applied in these post hoc tests to ensure statistical rigor by reducing Type I errors when evaluating group differences. Higher haplotype vulnerability values indicate a greater risk of loss under combined climatic and anthropogenic stresses.

### 2.5 Hotspot Populations Identification

A composite scoring system was developed to identify *E. przewalskii* hotspot populations for targeted conservation. Using R v4.3.1, a binary assignment model was constructed based on three filtering criteria: (I) populations located in the top 30% of vulnerability (indicating highly vulnerable areas); (II) populations with haplotype numbers in the top 30% (indicating rich haplotype diversity); and (III) populations containing haplotypes in the top 30% of vulnerability (representing highly vulnerable haplotypes). Each criterion was assigned a value of 1 if met, and 0 otherwise, resulting in a composite score ranging from 0 to 3. Populations scoring 3 were classified as highest‐priority hotspots, those scoring 2 as medium‐priority hotspots, those scoring 1 as low‐priority hotspots, and those scoring 0 as non‐priority hotspots. Owing to the constraints of limited resources, conservation efforts should be prioritized sequentially, starting with the highest‐priority hotspots and extending to lower‐priority populations as resources permit.

## 3 Results

### 3.1 Spatial Patterns of Vulnerability of *E. przewalskii*

**FIGURE 2:**
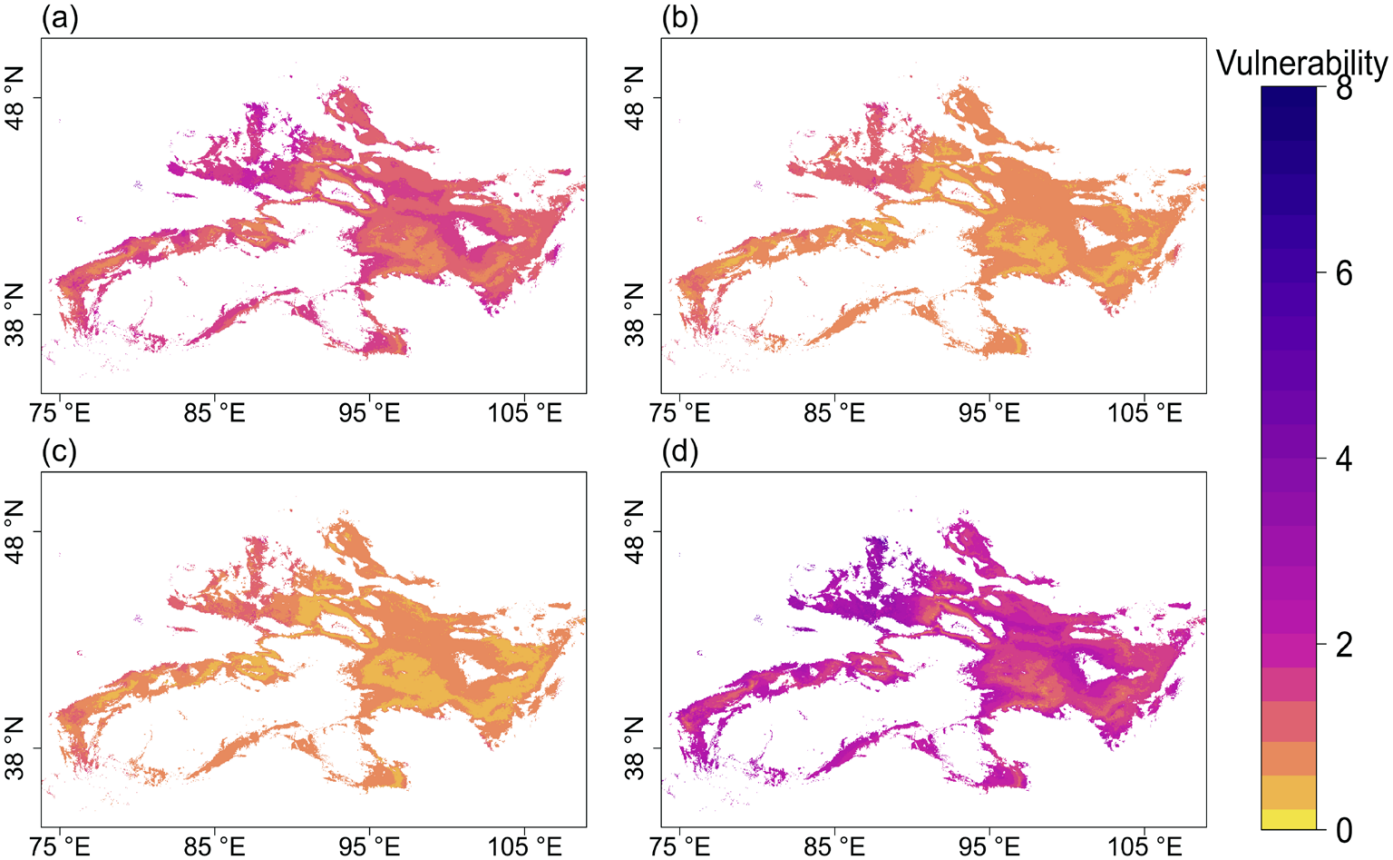
Spatial vulnerability of *Ephedra przewalskii* habitats under climatic and human activity variables. For ease of identification, the skewness of the color gradient was adjusted with a bias factor of 2.

*E. przewalskii* 's current potential distribution covered the core arid and semiarid regions, including the Junggar, Hami, and eastern Tarim Basins in Xinjiang (China), Umnugovi Province, and northern Cobado Province in Mongolia, with scattered habitat patches in the northeastern Tarim Basin and Altay Prefecture (Figure 2). Overall, the habitat vulnerability pattern exhibited an apparent “edge‐high, interior‐low” spatial gradient: high vulnerability was concentrated in the marginal areas of *E. przewalskii* 's distribution, whereas relatively low vulnerability occurred in its interior areas. Habitat vulnerability decreases in the 2050s as the SSP increased from 1–2.6 to 5–8.5 scenario. However, in the 2090s, vulnerability increases with higher SSPs. Under the fixed SSPs scenario, habitat vulnerability decreased over time under the SSP1‐2.6 scenario, but increased significantly in the 2090s under the SSP5‐8.5 scenario (Figure 2). Specifically, regions such as Nilka County, Hotan, Karamay, Wusu, and Shihezi City in Xinjiang, as well as Tuokexun County and Turpan City at the northwestern tip of Turpan, exhibited higher vulnerability under the 2050s‐SSP5‐8.5 and 2090‐SSP1‐2.6 scenarios. Similarly, areas such as Minqin County in Gansu, Zepu County, Shache County, Yecheng County, and Kashgar City in Xinjiang showed higher vulnerabilities (Figure 2b,c).

Although large areas exhibited high vulnerability under the 2050s‐SSP1‐2.6 and the 2090s‐SSP5‐8.5 scenarios, relatively low vulnerability occurred in the Ejina Banner‐Hami‐Heiying Mountain‐Jiuquan range area, Barkun Basin edge, and localized Tengger Desert regions (Figure 2a,d). Notably, under the combined climatic and anthropogenic impacts, habitat vulnerability did not increase with time or SSP intensification. However, vulnerability under the 2050s‐SSP5‐8.5 and the 2090s‐SSP1‐2.6 scenarios was lower than that under the 2050s‐SSP1‐2.6 scenario.

### 3.2 Risk of Haplotype Loss in *E. przewalskii*

**FIGURE 3:**
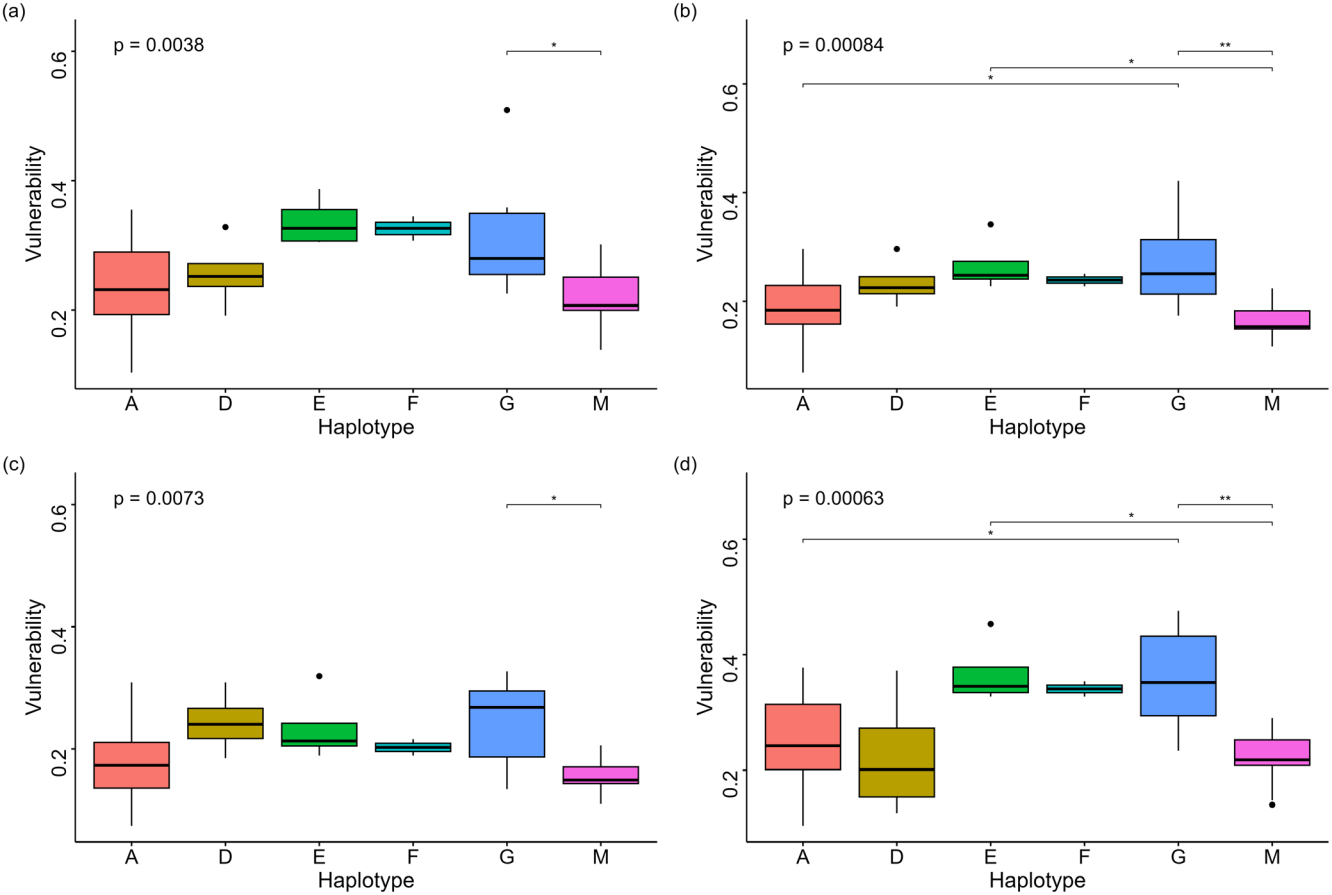
Comparison of vulnerability of haplogroups. Vulnerability values were normalized to a range of 0–1. (a) 2050s‐SSP1‐2.6 scenario; (b) 2050s‐SSP5‐8.5 scenario; (c) 2090s‐SSP1‐2.6 scenario; (d) 2090s‐SSP5‐8.5 scenario. *p*, overall ANOVA *p* value; *p*_adj_, pairwise *t*‐test *p* value (Bonferroni corrected); *p_adj_ < 0.05, **p_adj_ < 0.01.

The ANOVA results revealed significant differences in vulnerability (*p* < 0.05) among the haplotypes across all scenarios (Figure 3a–d). Multiple comparison tests revealed that haplotypes G and E exhibited higher vulnerability values in all four scenarios, whereas haplotypes A and M exhibited relatively lower vulnerability values. Haplotype G showed particularly pronounced differences compared with the others (Figure 3a–d). Under the SSP1‐2.6 scenario, only haplotypes G and M exhibited significant variability differences (*p*_adj_ < 0.05) (Figure 3a,c). In contrast, under the SSP5‐8.5 scenario, the vulnerability difference between G and M achieved a higher significance level (*p*_adj_ < 0.01), and statistically significant differences were observed between haplotypes E and M, as well as between haplotypes A and G (Figure 3b,d).

### 3.3 Hotspot Populations of *E. przewalskii*

**FIGURE 4:**
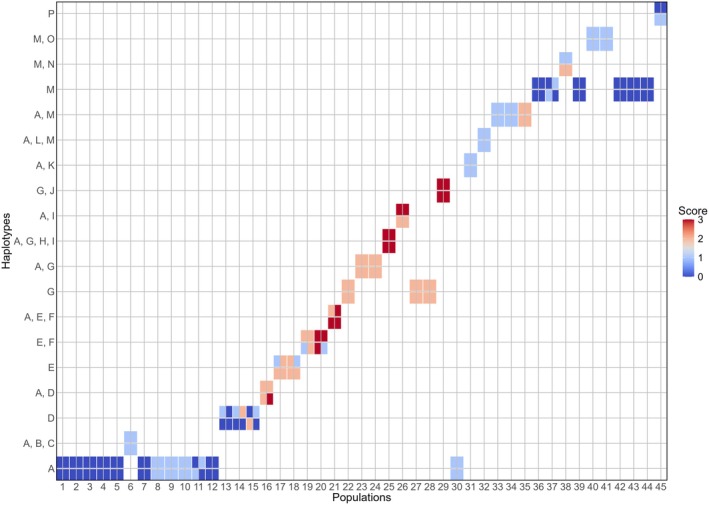
Biodiversity hotspots of *Ephedra przewalskii* under different scenarios. The four rectangular positions around the intersection represent four scenarios: (1) upper left: 2050s‐SSP1‐2.6 scenario; (2) upper right: 2050s‐SSP5‐8.5 scenario; (3) bottom left: 2090s‐SSP1‐2.6 scenario; (4) bottom right: 2090s‐SSP5‐8.5 scenario. Scores 0–3 in order of low‐priority, basic, sub‐priority, and high‐priority hotspots.

Of the 45 *E. przewalskii* populations, six (13.3%) were identified as highest‐priority hotspots (score = 3), concentrated in populations 16, 20, 21, 25, 26, and 29. These populations were distributed in highly vulnerable areas, exhibited rich haplotype diversity, and contained highly vulnerable haplotypes (e.g., E, F, G, H, and I) (Figure 4), with locations in the Tarim Basin (Wuheshalu, Qiemo, and Ruoqian) and the Junggar Basin (Karamay, Wuchang, and Burqin) (Table S2). Medium‐priority hotspots (score = 2), low‐priority hotspots (score = 1), and non‐hotspots (score = 0) accounted for 33.3%, 48.9%, and 40.0%, respectively, of the 45 populations. Most did not reach the highest rank owing to missing one or more indicators (insufficient haplotype numbers and lack of highly vulnerable haplotypes), but still exhibited localized aggregation (e.g., haplotypes B, C, K, M, O, N, P, and L in populations 1–15 and 30–45) (Figure 4). Notably, populations 14–29, 35, and 38 had higher scores than other populations. Populations 14–29 showed significant clustering, primarily concentrated in the Tarim Basin (Patrul, Atushi, Mayikake, Wuheshalu, Pishan, Cele, Qiemo, and Ruoqiang) and Junggar Basin (Qitai, Karamay, Wuchang, Beitashan, Alashankou, Hefeng, and Burqin) (Figure 4 and Table S2). Populations 35 and 38, located in the Alxa Desert (Ejina area) and the Hexi Corridor (Subei area), respectively, were identified as low‐priority hotspots and medium‐priority hotspots across all four scenarios (Figure 4 and Table S2).

## 4 Discussion

### 4.1 Habitat Vulnerability of *E. przewalskii*

*E. przewalskii* 's habitat vulnerability exhibited complex nonlinear changes over time and SSPs rather than a monotonic increase with SSP magnitude or time. Under combined climatic and human impacts, habitat vulnerability was generally lower under the 2050s‐SSP5‐8.5 and 2090s‐SSP1‐2.6 than under the 2050s‐SSP1‐2.6 and 2090s‐SSP5‐8.5. Notably, the mechanisms driving this nonlinear pattern lack direct species‐specific evidence and thus require further investigation. Over time, sustained high‐emissions result in notable climatic changes, including increasing temperatures, increased aridity, and more frequent and intense extreme weather events (Zhang, Zhang, and Ning 2024). These changes may degrade habitats by causing soil drought and alter vegetation structure, threatening long‐term survival (Li et al. 2017; Deng et al. 2022; Shi et al. 2023; Z. Wang 2024). By the 2090s, prolonged high CO_2_ under SSP5‐8.5 may saturate the plant's physiological adaptive capacity, preventing further benefits from increased CO_2_ levels. Additionally, climate change and anthropogenic activities may exceed the adaptive capacity of plants and influence a substantial increase in habitat vulnerability. Continued high emissions may also cause soil salinization and water quality deterioration, further affecting the growth and reproduction of *E. przewalskii* (Zhang, Chang, et al. 2015; Zhang, Li, et al. 2015).

### 4.2 Haplotype‐Specific Genetic Vulnerability of *E. przewalskii* : Risk Hierarchies and Conservation Priorities

Extreme climatic events, intensified by climate change and anthropogenic activities, present a major threat to plant genetic diversity (Hou et al. 2024). Increased focus should be given to haplotype populations exhibiting high genetic diversity to maintain species genetic richness under environmental stress. The study revealed that increasing climate forcing restructures haplotype risk hierarchies, from a singular gradient (G > M) under SSP1‐2.6 to a multilayered cascade (E > M; G > A > M) under SSP5‐8.5. Specifically, under a high‐emissions scenario, intensified climate change may further amplify the differences in vulnerability among haplotypes, potentially exerting more complex impacts on *E. przewalskii* 's ecological adaptation and population stability. Notably, all samples carrying haplotype G originated from the Jungar Basin (e.g., Karamay, Alashankou, Hefeng, and Burqin) and the Tarim Basin (e.g., Qitai1 and Beitashan), indicating that future conservation strategies should focus on *E. przewalskii* populations in these regions to prevent genetic diversity loss (Table S2). Although haplotype M populations in the Alashan Desert, Hexi Corridor, and Qaidam Basin showed lower vulnerability than haplotype G populations, the risk of genetic diversity loss in these areas may increase with climate extremes and should not be overlooked. Additionally, haplotype E in Pishan, Cele, Ruoqiang, and Qiemo Counties demonstrated significantly different vulnerability characteristics from haplotype M under SSP5‐8.5, indicating that these areas are also priorities for conservation. In contrast, the wider distribution of haplotype A may explain its lower risk of loss. Variations in vulnerability among haplotype populations result from differences in adaptability to environmental changes (Sang et al. 2022). This study revealed a significant geographical heterogeneity in *E. przewalskii* populations' responses to environmental changes driven by climate change and anthropogenic activities. Targeted conservation measures, such as establishing nature reserves in areas at high risk of loss, artificial breeding, and relocation conservation, can effectively preserve the genetic diversity of different haplotypes. These findings are crucial for guiding future conservation strategies for *E. przewalskii* and its genetic diversity.

Based on the vulnerability results of the potential distribution areas identified by the integrated species distribution models and CNFA, combined with the site‐specific vulnerability assessment of *E. przewalskii* 's 45 known populations, we constructed a multidimensional scoring system to assess population conservation priorities. Based on the ranking of scores for each population, we recommend implementing a stratified conservation strategy for *E. przewalskii* . First, priority should be given to conserving populations located in highly vulnerable areas, exhibiting rich haplotype diversity and containing highly vulnerable haplotypes. These populations are critical for maintaining the genetic diversity of the species and adapting to future environmental changes. Once high‐priority hotspots are effectively conserved, lower‐level targets (e.g., medium‐priority hotspots) can be addressed if additional resources are available. This hierarchical strategy maximizes limited resource efficiency while mitigating the risk of species extinction owing to habitat loss or environmental changes. Through scientific and systematic assessments and planning, we can provide more comprehensive, precise, and long‐term conservation measures for *E. przewalskii* , thereby promoting population health and ecosystem stability.

### 4.3 Study Limitations and Future Directions

Despite enhancing the accuracy of *E. przewalskii* distribution simulations and genetic vulnerability assessments via model integration and the inclusion of anthropogenic factors, this study has several limitations. First, future‐oriented projections retain modest uncertainties; variability across GCMs introduces inherent ambiguity (Figures S3 and S4), and human impact assessments rely on current activity patterns from the Global Human Modification dataset (owing to the unavailability of future dynamic anthropogenic data), which limits the ability to completely capture evolving human pressures. Second, the multidimensional vulnerability framework uses equal criterion weighting (neutral, insufficient evidence for differential ecological impacts), although it avoids overemphasizing a single factor without an ecological basis. Third, although spatial thinning and collinearity filtering mitigated spatial autocorrelation (SAC) at the input stage (Aiello Lammens et al. 2015; Dares 2019), residual diagnostics showed weak but significant SAC in each of the four individual ensemble models (Moran's *I*: 0.012–0.026, *p*_adj_ < 0.05, Table S3), whereas CEM showed nonsignificant residual SAC (Moran's *I*: −0.0040 to −0.0033, *p*_adj_ > 0.05; Table S3). Finally, nonclimatic variables (soil properties and biotic interactions) were excluded to avoid excessive multicollinearity and focus on our primary goal of quantifying the relative impacts of climate change and human activity on *E. przewalskii* haplotype vulnerability, although this overlooks secondary but ecologically relevant habitat constraints. Based on this study, future research should: integrate predictive anthropogenic datasets such as the Land‐Use Harmonization (LUH2; Hurtt et al. 2020), which enables linking land‐use patterns, agricultural management information, and climate scenarios; incorporate spatial autocorrelation models (Spatial Autoregressive Model) to further address residual SAC (Dormann et al. 2007); and supplement sampling for the 10 rare excluded haplotypes to validate the generalizability of vulnerability conclusions. Implementing these steps will enhance the comprehensiveness of projections while maintaining key insights into *E. przewalskii* 's genetic vulnerability under climate and human pressures.

## 5 Conclusions

Our study proposed a multidimensional scoring system to precisely identify and prioritize the protection of key areas with high haplotype diversity and vulnerability, thereby optimizing resource utilization and providing an effective adaptive tool for assessing the vulnerability of drought‐adapted species. These findings evidently indicate that populations of *E. przewalskii* in the Tarim Basin (Wuheshalu, Qiemo, and Ruoqian) and Junggar Basin (Karamay, Wuchang, and Burqin) should be prioritized for conservation efforts. Furthermore, this study revealed how climate change and human activities exacerbate the risks of habitat fragmentation, loss, and genetic diversity decline, underscoring the urgency of implementing comprehensive conservation measures.

## Author Contributions

**Hongchao Wang:** data curation (equal), formal analysis (equal), writing – original draft (equal), writing – review and editing (equal). **Qi'ao Ma:** data curation (equal), validation (equal), writing – review and editing (equal). **Liwei Wang:** data curation (equal), validation (equal), writing – review and editing (equal). **Ying Li:** writing – review and editing (equal). **Meng Cheng:** writing – review and editing (equal). **Xiaolin Li:** writing – review and editing (equal). **Guang Yang:** writing – review and editing (equal). **Kai Sun:** conceptualization (equal), supervision (equal), writing – review and editing (equal). **Xiulian Chi:** conceptualization (equal), supervision (equal), writing – review and editing (equal).

## Funding

This work was supported by the National Natural Science Foundation of China (82173930), the Scientific and Technological Innovation Project of China Academy of Chinese Medical Sciences (CI2023E002), the Science and Technology Fundamental Resources Investigation Program (2023FY100700, 2023FY100701‐6), and the Fundamental Research Funds for the Central public welfare research institutes (ZZ13‐YQ‐087).

## Conflicts of Interest

The authors declare no conflicts of interest.

## Supporting information


**Figure S1:** Selection of climate variables. (a) Adjusted *D* ^2^ of the logistic model and Maxent projected contribution of each variable; (b) Variable correlation heat map.
**Figure S2:** Rarefaction curve for six target haplotypes (*n* ≥ 3 sampling points). The dashed red line indicates the total sampling point positions; the dashed orange line indicates the start of the plateau phase. The curve plateaued at 95% of expected individual richness with Good's coverage ≥ 0.95, indicating that for these six core haplotypes, the existing number of sampling points (≥ 19) is sufficient to indicate their overall distribution patterns.
**Figure S3:** Spatial coefficient of variation of *Ephedra przewalskii* habitat vulnerability across three GCMs.
**Figure S4:** Spatial 95% confidence intervals of *Ephedra przewalskii* habitat vulnerability under combined climate and human activity drivers.
**Table S1:** Wild geographic distribution points of *Ephedra przewalskii*.
**Table S2:** Details of sample locations, sample size, and haplotype frequencies for 45 populations of *Ephedra przewalskii* . Figures in parentheses represent the number of the haplotypes (adapted from Su and Zhang 2016).
**Table S3:** Spatial autocorrelation tests of ensemble predictions under three pseudo‐absence replicates.

## Data Availability

The data that support the findings of this study are available in the Supporting Information of this article.
