# Supplementary material for: Habitat and Haplotype‐Specific Genetic Vulnerability Analysis Combined With a Multidimensional Scoring System Provides a New Insight for Conservation Prioritization of Ephedra przewalskii
Source: Ecol Evol. 2025 Dec 14;15(12):e72614. doi: 10.1002/ece3.72614 (PMC12703116; doi:10.1002/ece3.72614)
Supplement: Supplementary file 1 — Figure S1: Selection of climate variables. (a) Adjusted D 2 of the logistic model and Maxent projected contribution of each variable; (b) Variable correlation heat map. Figure S2: Rarefaction curve for six target haplotypes (n ≥ 3 sampling points). The dashed red line indicates the total sampling point positions; the dashed orange line indicates the start of the plateau phase. The curve plateaued at 95% of expected individual richness with Good's coverage ≥ 0.95, indicating that for these six core haplotypes, the existing number of sampling points (≥ 19) is sufficient to indicate their overall distribution patterns. Figure S3: Spatial coefficient of variation of Ephedra przewalskii habitat vulnerability across three GCMs. Figure S4: Spatial 95% confidence intervals of Ephedra przewalskii habitat vulnerability under combined climate and human activity drivers. Table S1: Wild geographic distribution points of Ephedra przewalskii. Table S2: Details of sample locations, sample size, and haplotype frequencies for 45 populations of Ephedra przewalskii . Figures in parentheses represent the number of the haplotypes (adapted from Su and Zhang 2016). Table S3: Spatial autocorrelation tests of ensemble predictions under three pseudo‐absence replicates. [file ECE3-15-e72614-s001.docx]

**
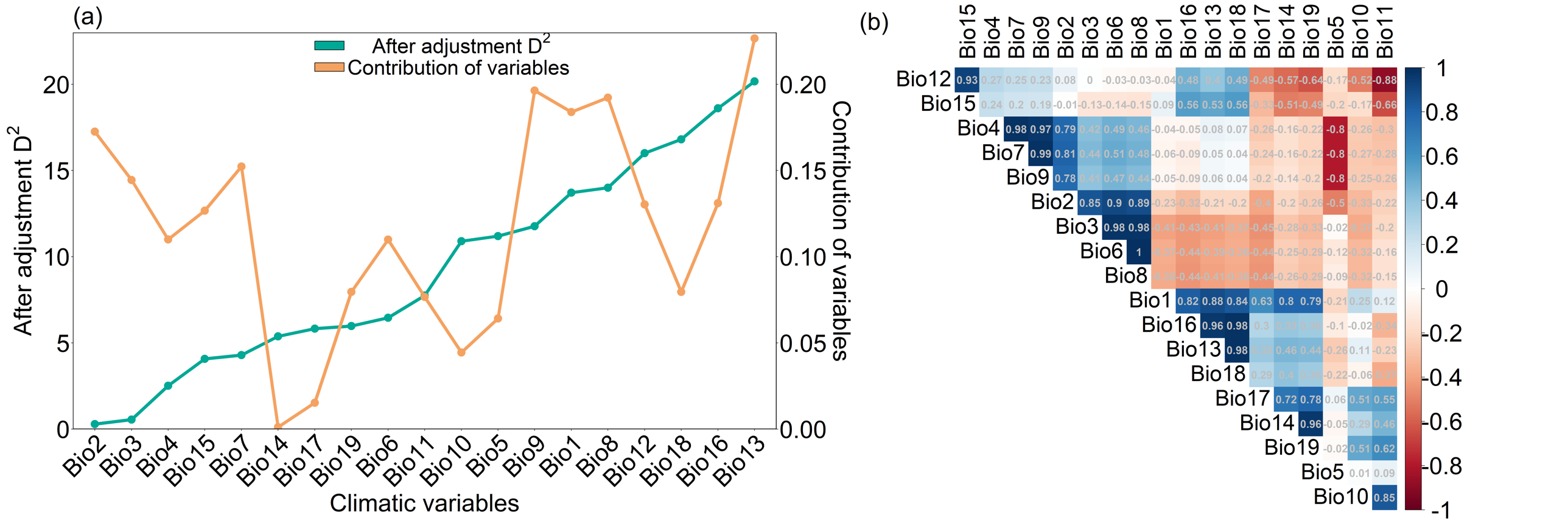
**

**Figure S1** | Selection of climate variables. (a) Adjusted D2 of the logistic model and Maxent projected contribution of each variable; (b) Variable correlation heat map.


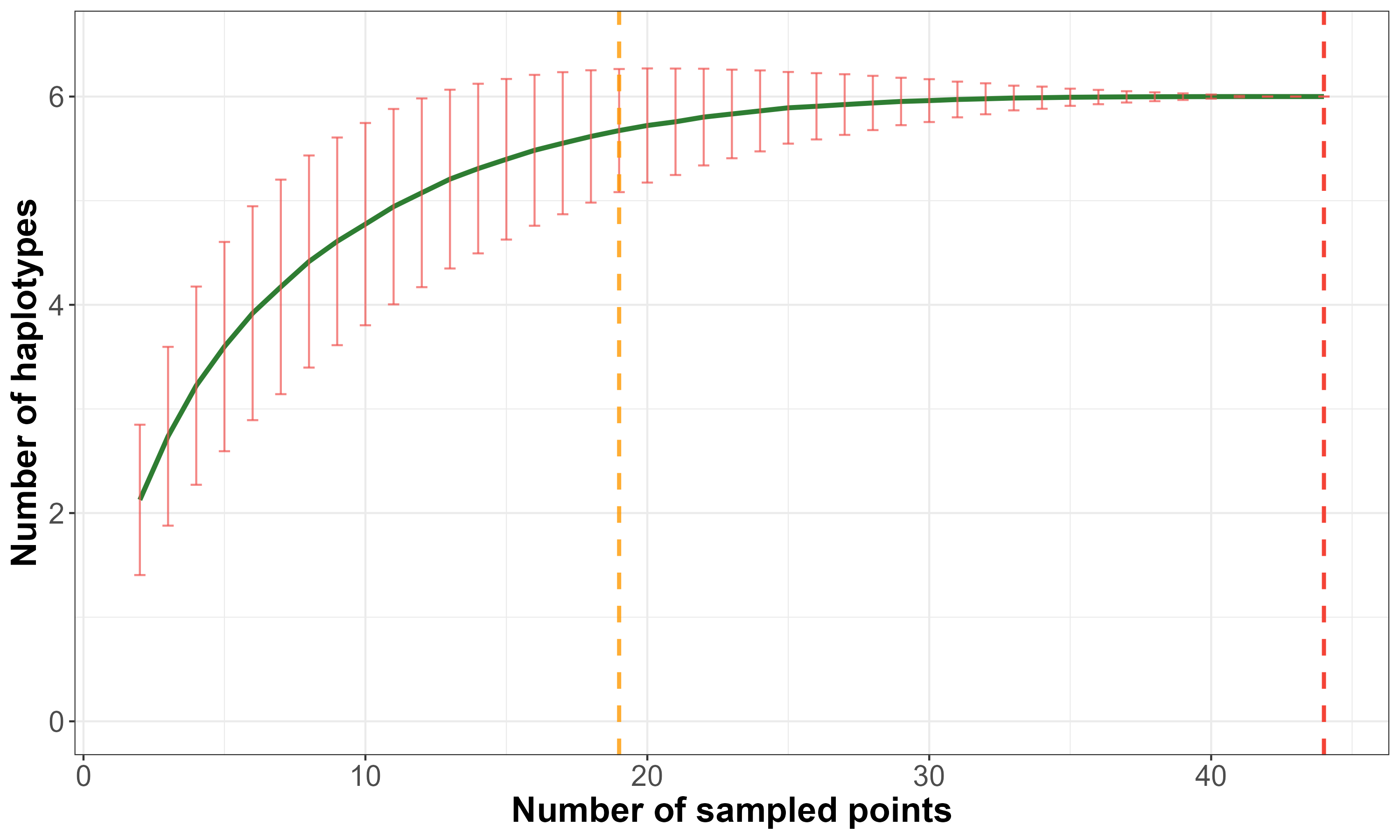


**Figure S2 |** Rarefaction curve for six target haplotypes (n≥3 sampling points). The dashed red line indicates the total sampling point positions; the dashed orange line indicates the start of the plateau phase. The curve plateaued at 95 % of expected individual richness with Good’s coverage ≥ 0.95, indicating that for these six core haplotypes, the existing number of sampling points (≥19) is sufficient to indicate their overall distribution patterns.


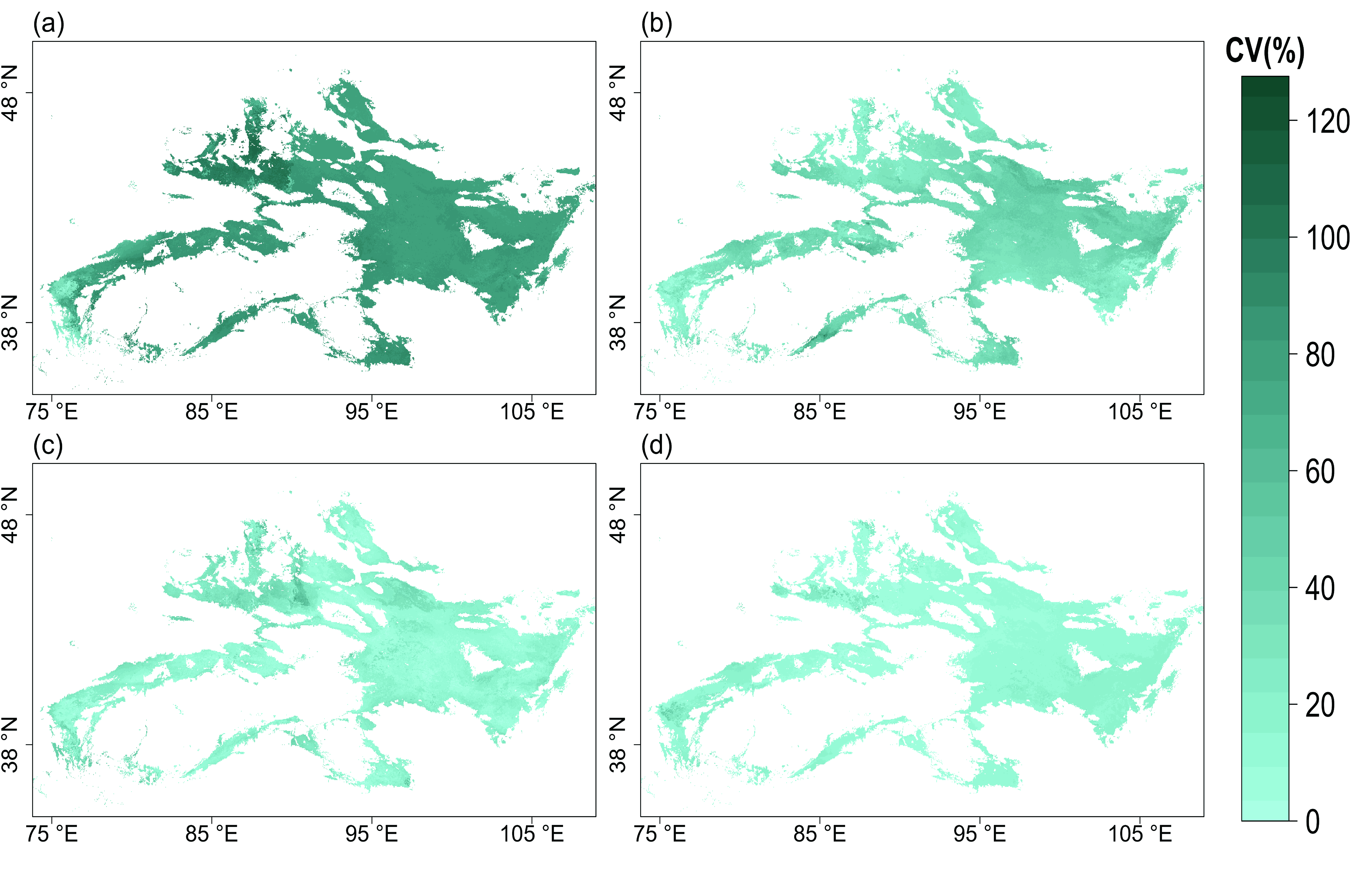


**Figure S3** | Spatial coefficient of variation of E. przewalskii habitat vulnerability across three GCMs.


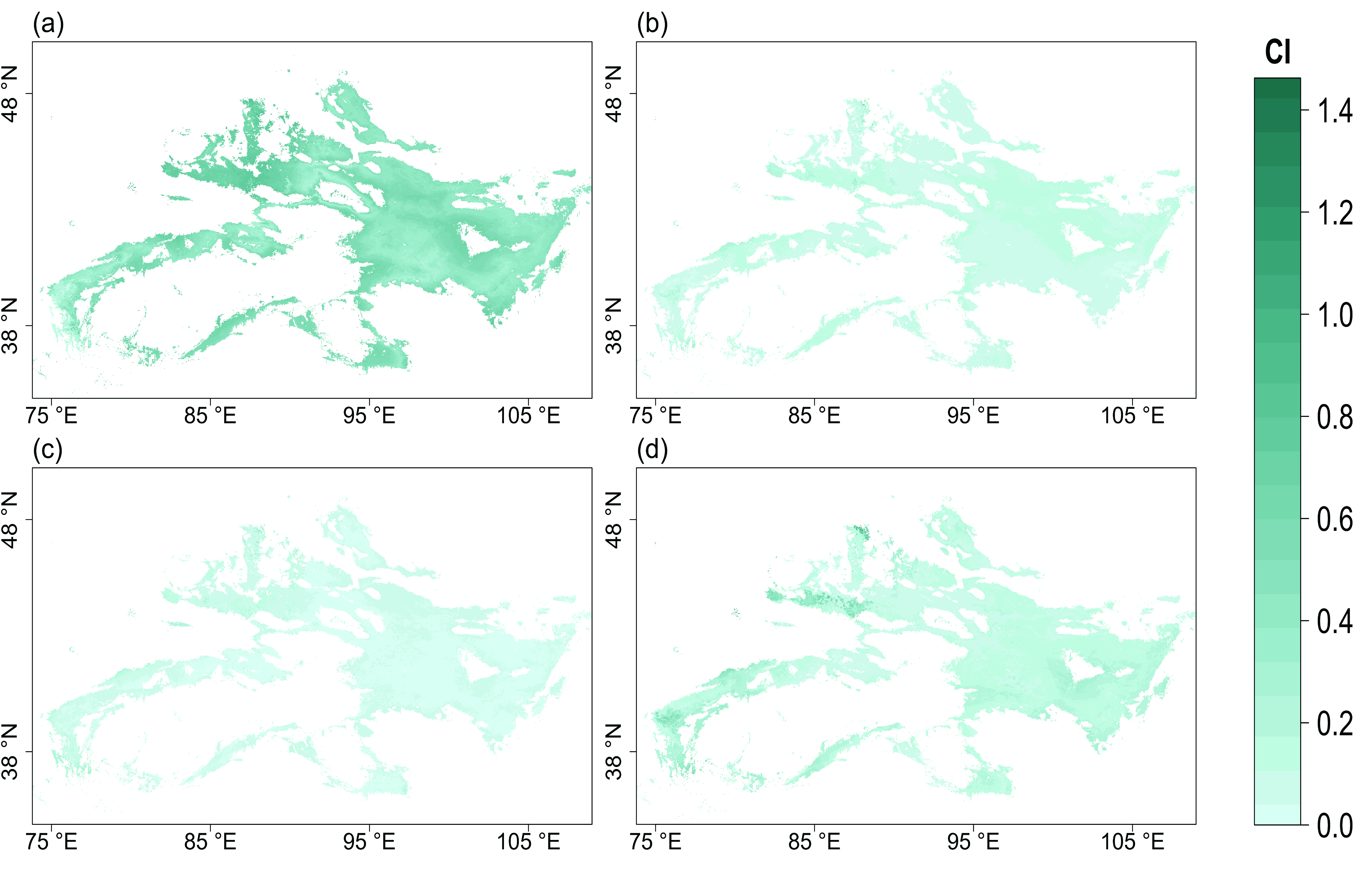


**Figure S4** | Spatial 95% confidence intervals of *E. przewalskii* habitat vulnerability under combined climate and human activity drivers.

**TABLE S1** | Wild geographic distribution points of *Ephedra przewalskii.*

| Species | SourceID | Year | Lon | Lat |
| --- | --- | --- | --- | --- |
| *Ephedra przewalskii* | Alus,2021 | 2021 | 106.631294 | 41.974422 |
| *Ephedra przewalskii* | CAS PSDC, https://www.plantplus.cn/ | 2024 | 97.6327 | 39.927817 |
| *Ephedra przewalskii* | CAS PSDC, https://www.plantplus.cn/ | 2024 | 98.337322 | 40.491792 |
| *Ephedra przewalskii* | CAS PSDC, https://www.plantplus.cn/ | 2024 | 98.341725 | 40.515572 |
| *Ephedra przewalskii* | CAS PSDC, https://www.plantplus.cn/ | 2024 | 98.342519 | 40.809378 |
| *Ephedra przewalskii* | CAS PSDC, https://www.plantplus.cn/ | 2024 | 98.356575 | 40.867303 |
| *Ephedra przewalskii* | CAS PSDC, https://www.plantplus.cn/ | 2024 | 98.433308 | 42.032333 |
| *Ephedra przewalskii* | CAS PSDC, https://www.plantplus.cn/ | 2024 | 98.444733 | 42.181039 |
| *Ephedra przewalskii* | CAS PSDC, https://www.plantplus.cn/ | 2024 | 98.446786 | 42.0994 |
| *Ephedra przewalskii* | CAS PSDC, https://www.plantplus.cn/ | 2024 | 98.508233 | 42.164406 |
| *Ephedra przewalskii* | CAS PSDC, https://www.plantplus.cn/ | 2024 | 98.508694 | 42.164294 |
| *Ephedra przewalskii* | CAS PSDC, https://www.plantplus.cn/ | 2024 | 98.651611 | 42.170186 |
| *Ephedra przewalskii* | CAS PSDC, https://www.plantplus.cn/ | 2024 | 98.830267 | 42.136322 |
| *Ephedra przewalskii* | CAS PSDC, https://www.plantplus.cn/ | 2024 | 98.901603 | 42.146764 |
| *Ephedra przewalskii* | CAS PSDC, https://www.plantplus.cn/ | 2024 | 99.208028 | 40.315322 |
| *Ephedra przewalskii* | CAS PSDC, https://www.plantplus.cn/ | 2024 | 100.409406 | 41.045331 |
| *Ephedra przewalskii* | CAS PSDC, https://www.plantplus.cn/ | 2024 | 100.899358 | 41.944631 |
| *Ephedra przewalskii* | CVH, http://www.cvh.org.cn/cms/ | 2018 | 75.03 | 39.72 |
| *Ephedra przewalskii* | CVH, http://www.cvh.org.cn/cms/ | 2007 | 76.06116667 | 38.74616667 |
| *Ephedra przewalskii* | CVH, http://www.cvh.org.cn/cms/ | 2016 | 82.66 | 44.14 |
| *Ephedra przewalskii* | CVH, http://www.cvh.org.cn/cms/ | 2007 | 83.65 | 41.79 |
| *Ephedra przewalskii* | CVH, http://www.cvh.org.cn/cms/ | 2007 | 85.74 | 41.85 |
| *Ephedra przewalskii* | CVH, http://www.cvh.org.cn/cms/ | 2007 | 86.89 | 42.29 |
| *Ephedra przewalskii* | CVH, http://www.cvh.org.cn/cms/ | 2007 | 88.28 | 44.8 |
| *Ephedra przewalskii* | CVH, http://www.cvh.org.cn/cms/ | 2007 | 88.48 | 44.95 |
| *Ephedra przewalskii* | CVH, http://www.cvh.org.cn/cms/ | 2017 | 90.11 | 45.2 |
| *Ephedra przewalskii* | CVH, http://www.cvh.org.cn/cms/ | 2016 | 90.81 | 38.42 |
| *Ephedra przewalskii* | CVH, http://www.cvh.org.cn/cms/ | 2002 | 94.7 | 38.25 |
| *Ephedra przewalskii* | CVH, http://www.cvh.org.cn/cms/ | 2006 | 94.80125 | 43.26385 |
| *Ephedra przewalskii* | CVH, http://www.cvh.org.cn/cms/ | 2011 | 94.81 | 36.43 |
| *Ephedra przewalskii* | CVH, http://www.cvh.org.cn/cms/ | 2002 | 95.08 | 38.02 |
| *Ephedra przewalskii* | CVH, http://www.cvh.org.cn/cms/ | 2008 | 95.13 | 41.8 |
| *Ephedra przewalskii* | CVH, http://www.cvh.org.cn/cms/ | 2008 | 95.14 | 41.81 |
| *Ephedra przewalskii* | CVH, http://www.cvh.org.cn/cms/ | 2008 | 95.14 | 41.75 |
| *Ephedra przewalskii* | CVH, http://www.cvh.org.cn/cms/ | 2008 | 95.15 | 41.82 |
| *Ephedra przewalskii* | CVH, http://www.cvh.org.cn/cms/ | 2008 | 95.17 | 41.84 |
| *Ephedra przewalskii* | CVH, http://www.cvh.org.cn/cms/ | 2008 | 95.18 | 41.86 |
| *Ephedra przewalskii* | CVH, http://www.cvh.org.cn/cms/ | 2008 | 95.2 | 41.69 |
| *Ephedra przewalskii* | CVH, http://www.cvh.org.cn/cms/ | 2008 | 95.25 | 41.51 |
| *Ephedra przewalskii* | CVH, http://www.cvh.org.cn/cms/ | 2008 | 95.31 | 41.61 |
| *Ephedra przewalskii* | CVH, http://www.cvh.org.cn/cms/ | 2018 | 96.09 | 36.37 |
| *Ephedra przewalskii* | CVH, http://www.cvh.org.cn/cms/ | 2018 | 96.56 | 40.43 |
| *Ephedra przewalskii* | CVH, http://www.cvh.org.cn/cms/ | 2017 | 96.64 | 37.31 |
| *Ephedra przewalskii* | CVH, http://www.cvh.org.cn/cms/ | 2002 | 97.46 | 36.06 |
| *Ephedra przewalskii* | CVH, http://www.cvh.org.cn/cms/ | 2017 | 97.83 | 41.5 |
| *Ephedra przewalskii* | CVH, http://www.cvh.org.cn/cms/ | 2017 | 97.95 | 41.36 |
| *Ephedra przewalskii* | CVH, http://www.cvh.org.cn/cms/ | 2017 | 98.02 | 41.39 |
| *Ephedra przewalskii* | CVH, http://www.cvh.org.cn/cms/ | 2017 | 98.06 | 41.25 |
| *Ephedra przewalskii* | CVH, http://www.cvh.org.cn/cms/ | 2017 | 98.12 | 41.54 |
| *Ephedra przewalskii* | CVH, http://www.cvh.org.cn/cms/ | 2017 | 98.58 | 39.67 |
| *Ephedra przewalskii* | CVH, http://www.cvh.org.cn/cms/ | 2017 | 99.29 | 40.38 |
| *Ephedra przewalskii* | CVH, http://www.cvh.org.cn/cms/ | 2017 | 99.65 | 42.04 |
| *Ephedra przewalskii* | CVH, http://www.cvh.org.cn/cms/ | 2017 | 99.7 | 40.97 |
| *Ephedra przewalskii* | CVH, http://www.cvh.org.cn/cms/ | 2017 | 99.9 | 41.55 |
| *Ephedra przewalskii* | CVH, http://www.cvh.org.cn/cms/ | 2017 | 100.27 | 42.61 |
| *Ephedra przewalskii* | CVH, http://www.cvh.org.cn/cms/ | 2017 | 100.55 | 41.55 |
| *Ephedra przewalskii* | CVH, http://www.cvh.org.cn/cms/ | 2017 | 100.57 | 41.23 |
| *Ephedra przewalskii* | CVH, http://www.cvh.org.cn/cms/ | 2017 | 100.62 | 41.6 |
| *Ephedra przewalskii* | CVH, http://www.cvh.org.cn/cms/ | 2017 | 100.69 | 41.76 |
| *Ephedra przewalskii* | CVH, http://www.cvh.org.cn/cms/ | 2017 | 100.84 | 40.25 |
| *Ephedra przewalskii* | CVH, http://www.cvh.org.cn/cms/ | 2017 | 100.95 | 40.84 |
| *Ephedra przewalskii* | CVH, http://www.cvh.org.cn/cms/ | 2017 | 100.96 | 42.65 |
| *Ephedra przewalskii* | CVH, http://www.cvh.org.cn/cms/ | 2017 | 100.97 | 41.17 |
| *Ephedra przewalskii* | CVH, http://www.cvh.org.cn/cms/ | 2016 | 102.1 | 41.99 |
| *Ephedra przewalskii* | CVH, http://www.cvh.org.cn/cms/ | 2017 | 102.89 | 39.47 |
| *Ephedra przewalskii* | GBIF, https://www.gbif.org/ | 2016 | 87.6 | 43.7 |
| *Ephedra przewalskii* | GBIF, https://www.gbif.org/ | 2012 | 87.983 | 47.633 |
| *Ephedra przewalskii* | GBIF, https://www.gbif.org/ | 2019 | 91.686652 | 45.244276 |
| *Ephedra przewalskii* | GBIF, https://www.gbif.org/ | 2019 | 91.799369 | 45.168807 |
| *Ephedra przewalskii* | GBIF, https://www.gbif.org/ | 2019 | 92.046455 | 45.159124 |
| *Ephedra przewalskii* | GBIF, https://www.gbif.org/ | 2020 | 92.360113 | 45.405502 |
| *Ephedra przewalskii* | GBIF, https://www.gbif.org/ | 2004 | 92.376908 | 47.492884 |
| *Ephedra przewalskii* | GBIF, https://www.gbif.org/ | 2023 | 92.825814 | 45.4036 |
| *Ephedra przewalskii* | GBIF, https://www.gbif.org/ | 2023 | 93.122453 | 45.339733 |
| *Ephedra przewalskii* | GBIF, https://www.gbif.org/ | 2023 | 93.128372 | 45.3382 |
| *Ephedra przewalskii* | GBIF, https://www.gbif.org/ | 2020 | 93.14167 | 45.808492 |
| *Ephedra przewalskii* | GBIF, https://www.gbif.org/ | 2021 | 93.284247 | 45.359647 |
| *Ephedra przewalskii* | GBIF, https://www.gbif.org/ | 2021 | 93.34575 | 45.450119 |
| *Ephedra przewalskii* | GBIF, https://www.gbif.org/ | 2021 | 93.479294 | 49.035033 |
| *Ephedra przewalskii* | GBIF, https://www.gbif.org/ | 2017 | 93.619496 | 46.935591 |
| *Ephedra przewalskii* | GBIF, https://www.gbif.org/ | 2020 | 93.622514 | 45.537731 |
| *Ephedra przewalskii* | GBIF, https://www.gbif.org/ | 2023 | 93.649697 | 45.5386 |
| *Ephedra przewalskii* | GBIF, https://www.gbif.org/ | 2023 | 93.654608 | 45.538892 |
| *Ephedra przewalskii* | GBIF, https://www.gbif.org/ | 2021 | 93.678719 | 45.552311 |
| *Ephedra przewalskii* | GBIF, https://www.gbif.org/ | 2020 | 94.338225 | 45.391483 |
| *Ephedra przewalskii* | GBIF, https://www.gbif.org/ | 2020 | 96.250122 | 44.933436 |
| *Ephedra przewalskii* | GBIF, https://www.gbif.org/ | 2023 | 96.722778 | 44.456389 |
| *Ephedra przewalskii* | GBIF, https://www.gbif.org/ | 2023 | 96.750833 | 44.3925 |
| *Ephedra przewalskii* | GBIF, https://www.gbif.org/ | 2023 | 96.772778 | 44.221944 |
| *Ephedra przewalskii* | GBIF, https://www.gbif.org/ | 2023 | 96.814167 | 43.871389 |
| *Ephedra przewalskii* | GBIF, https://www.gbif.org/ | 2023 | 96.833056 | 44.755278 |
| *Ephedra przewalskii* | GBIF, https://www.gbif.org/ | 2016 | 97.177489 | 45.70484 |
| *Ephedra przewalskii* | GBIF, https://www.gbif.org/ | 2023 | 97.324722 | 43.289444 |
| *Ephedra przewalskii* | GBIF, https://www.gbif.org/ | 2023 | 97.341111 | 43.292778 |
| *Ephedra przewalskii* | GBIF, https://www.gbif.org/ | 2023 | 97.571667 | 43.283056 |
| *Ephedra przewalskii* | GBIF, https://www.gbif.org/ | 2023 | 97.6375 | 43.29 |
| *Ephedra przewalskii* | GBIF, https://www.gbif.org/ | 2023 | 97.723056 | 43.273056 |
| *Ephedra przewalskii* | GBIF, https://www.gbif.org/ | 2023 | 97.779444 | 43.296111 |
| *Ephedra przewalskii* | GBIF, https://www.gbif.org/ | 2023 | 97.785833 | 43.308056 |
| *Ephedra przewalskii* | GBIF, https://www.gbif.org/ | 2023 | 97.858781 | 43.21358 |
| *Ephedra przewalskii* | GBIF, https://www.gbif.org/ | 2023 | 97.861389 | 43.207222 |
| *Ephedra przewalskii* | GBIF, https://www.gbif.org/ | 2023 | 98.059444 | 43.152222 |
| *Ephedra przewalskii* | GBIF, https://www.gbif.org/ | 2023 | 98.11 | 44.452222 |
| *Ephedra przewalskii* | GBIF, https://www.gbif.org/ | 2023 | 98.4525 | 42.998333 |
| *Ephedra przewalskii* | GBIF, https://www.gbif.org/ | 2023 | 98.547778 | 43.864444 |
| *Ephedra przewalskii* | GBIF, https://www.gbif.org/ | 2024 | 98.781944 | 43.066944 |
| *Ephedra przewalskii* | GBIF, https://www.gbif.org/ | 2023 | 98.850278 | 42.915 |
| *Ephedra przewalskii* | GBIF, https://www.gbif.org/ | 2023 | 98.856944 | 42.927222 |
| *Ephedra przewalskii* | GBIF, https://www.gbif.org/ | 2023 | 98.945278 | 43.700833 |
| *Ephedra przewalskii* | GBIF, https://www.gbif.org/ | 2023 | 98.9525 | 43.096944 |
| *Ephedra przewalskii* | GBIF, https://www.gbif.org/ | 2022 | 99.051745 | 43.315394 |
| *Ephedra przewalskii* | GBIF, https://www.gbif.org/ | 2023 | 99.0575 | 43.322222 |
| *Ephedra przewalskii* | GBIF, https://www.gbif.org/ | 2022 | 99.466438 | 45.645533 |
| *Ephedra przewalskii* | GBIF, https://www.gbif.org/ | 2024 | 99.812636 | 43.472783 |
| *Ephedra przewalskii* | GBIF, https://www.gbif.org/ | 2024 | 99.870339 | 43.459142 |
| *Ephedra przewalskii* | GBIF, https://www.gbif.org/ | 2024 | 100.662658 | 43.021642 |
| *Ephedra przewalskii* | GBIF, https://www.gbif.org/ | 2024 | 101.062042 | 43.503964 |
| *Ephedra przewalskii* | GBIF, https://www.gbif.org/ | 2021 | 101.223542 | 42.942078 |
| *Ephedra przewalskii* | GBIF, https://www.gbif.org/ | 2023 | 101.312209 | 42.592882 |
| *Ephedra przewalskii* | GBIF, https://www.gbif.org/ | 2022 | 102.585861 | 43.86475 |
| *Ephedra przewalskii* | GBIF, https://www.gbif.org/ | 2024 | 102.602513 | 43.898038 |
| *Ephedra przewalskii* | GBIF, https://www.gbif.org/ | 2020 | 103.08 | 38.62 |
| *Ephedra przewalskii* | GBIF, https://www.gbif.org/ | 2006 | 105.67 | 38.83 |
| *Ephedra przewalskii* | GBIF, https://www.gbif.org/ | 2022 | 106.107795 | 42.181367 |
| *Ephedra przewalskii* | GBIF, https://www.gbif.org/ | 2022 | 106.142189 | 42.55975 |
| *Ephedra przewalskii* | GBIF, https://www.gbif.org/ | 2019 | 107.957292 | 43.085019 |
| *Ephedra przewalskii* | Han et al., 2024 | 2024 | 87.4 | 45.4 |
| *Ephedra przewalskii* | Li, 2020 | 2018 | 95.15 | 41.333333 |
| *Ephedra przewalskii* | Meng et al., 2022; | 2012 | 88.416667 | 44.566667 |
| *Ephedra przewalskii* | Pironon，2024 | 1993 | 96.17 | 36.37 |
| *Ephedra przewalskii* | Pironon，2024 | 1993 | 95.3 | 36.32 |
| *Ephedra przewalskii* | Pironon，2024 | 1999 | 94.91 | 36.36 |
| *Ephedra przewalskii* | Pironon，2024 | 1993 | 84.89 | 45.58 |
| *Ephedra przewalskii* | Su & Zhang, 2016 | 2015 | 74.75 | 39.66 |
| *Ephedra przewalskii* | Su & Zhang, 2016 | 2015 | 75.1 | 39.59 |
| *Ephedra przewalskii* | Su & Zhang, 2016 | 2015 | 76.2 | 39.73 |
| *Ephedra przewalskii* | Su & Zhang, 2016 | 2015 | 78.18 | 37.58 |
| *Ephedra przewalskii* | Su & Zhang, 2016 | 2015 | 78.44 | 39.96 |
| *Ephedra przewalskii* | Su & Zhang, 2016 | 2015 | 79.82 | 40.69 |
| *Ephedra przewalskii* | Su & Zhang, 2016 | 2015 | 80.82 | 36.97 |
| *Ephedra przewalskii* | Su & Zhang, 2016 | 2015 | 81.25 | 41.57 |
| *Ephedra przewalskii* | Su & Zhang, 2016 | 2015 | 81.65 | 41.85 |
| *Ephedra przewalskii* | Su & Zhang, 2016 | 2015 | 82.56 | 45.1 |
| *Ephedra przewalskii* | Su & Zhang, 2016 | 2015 | 82.77 | 41.85 |
| *Ephedra przewalskii* | Su & Zhang, 2016 | 2015 | 83.39 | 41.76 |
| *Ephedra przewalskii* | Su & Zhang, 2016 | 2015 | 84.15 | 41.95 |
| *Ephedra przewalskii* | Su & Zhang, 2016 | 2015 | 84.84 | 45.63 |
| *Ephedra przewalskii* | Su & Zhang, 2016 | 2015 | 85.36 | 45.89 |
| *Ephedra przewalskii* | Su & Zhang, 2016 | 2015 | 85.59 | 38.03 |
| *Ephedra przewalskii* | Su & Zhang, 2016 | 2015 | 85.86 | 46.24 |
| *Ephedra przewalskii* | Su & Zhang, 2016 | 2015 | 86.34 | 41.87 |
| *Ephedra przewalskii* | Su & Zhang, 2016 | 2015 | 86.92 | 47.65 |
| *Ephedra przewalskii* | Su & Zhang, 2016 | 2015 | 87.1 | 38.71 |
| *Ephedra przewalskii* | Su & Zhang, 2016 | 2015 | 87.43 | 42.23 |
| *Ephedra przewalskii* | Su & Zhang, 2016 | 2015 | 87.82 | 42.22 |
| *Ephedra przewalskii* | Su & Zhang, 2016 | 2015 | 88.15 | 39 |
| *Ephedra przewalskii* | Su & Zhang, 2016 | 2015 | 88.18 | 42.24 |
| *Ephedra przewalskii* | Su & Zhang, 2016 | 2015 | 88.35 | 43.3 |
| *Ephedra przewalskii* | Su & Zhang, 2016 | 2015 | 88.53 | 42.48 |
| *Ephedra przewalskii* | Su & Zhang, 2016 | 2015 | 89.63 | 44.25 |
| *Ephedra przewalskii* | Su & Zhang, 2016 | 2015 | 90.13 | 44.27 |
| *Ephedra przewalskii* | Su & Zhang, 2016 | 2015 | 90.35 | 44.6 |
| *Ephedra przewalskii* | Su & Zhang, 2016 | 2015 | 93.62 | 42.91 |
| *Ephedra przewalskii* | Su & Zhang, 2016 | 2015 | 94.36 | 39.67 |
| *Ephedra przewalskii* | Su & Zhang, 2016 | 2015 | 94.57 | 39.74 |
| *Ephedra przewalskii* | Su & Zhang, 2016 | 2015 | 94.8 | 36.29 |
| *Ephedra przewalskii* | Su & Zhang, 2016 | 2015 | 95.1 | 37.95 |
| *Ephedra przewalskii* | Su & Zhang, 2016 | 2015 | 95.38 | 41.17 |
| *Ephedra przewalskii* | Su & Zhang, 2016 | 2015 | 95.4 | 37.43 |
| *Ephedra przewalskii* | Su & Zhang, 2016 | 2015 | 95.5 | 37.2 |
| *Ephedra przewalskii* | Su & Zhang, 2016 | 2015 | 95.67 | 40.39 |
| *Ephedra przewalskii* | Su & Zhang, 2016 | 2015 | 95.68 | 37.58 |
| *Ephedra przewalskii* | Su & Zhang, 2016 | 2015 | 96.42 | 36.37 |
| *Ephedra przewalskii* | Su & Zhang, 2016 | 2015 | 97.06 | 40.22 |
| *Ephedra przewalskii* | Su & Zhang, 2016 | 2015 | 98.92 | 39.97 |
| *Ephedra przewalskii* | Su & Zhang, 2016 | 2015 | 99.81 | 40.35 |
| *Ephedra przewalskii* | Su & Zhang, 2016 | 2015 | 100.63 | 41.87 |
| *Ephedra przewalskii* | Su & Zhang, 2016 | 2015 | 102.86 | 38.57 |
| *Ephedra przewalskii* | Ma et al.,2018 | 2012 | 86.266667 | 42.483333 |
| *Ephedra przewalskii* | Ma et al.,2018 | 2012 | 86.35 | 42.316667 |
| *Ephedra przewalskii* | Ma et al.,2018 | 2012 | 86.9 | 42.283333 |
| *Ephedra przewalskii* | Ma et al.,2018 | 2012 | 88.483333 | 42.3 |
| *Ephedra przewalskii* | Ma et al.,2018 | 2012 | 91.566667 | 43.366667 |
| *Ephedra przewalskii* | Xing & Zhang, 2021 | 2021 | 76.88124847 | 37.07310867 |
| *Ephedra przewalskii* | Zhang et al., 2015 | 2012 | 103.419722 | 38.953056 |
| *Ephedra przewalskii* | Zhang et al., 2015 | 2012 | 102.969722 | 38.954722 |

**TABLE S2** | Details of sample locations, sample size, and haplotype frequencies for 45 populations of *E. przewalskii*. Figures in parentheses represent the number of the haplotypes. (Adapted from Su and Zhang, 2016)

| Region | Number | Location | Cp haplotype |
| --- | --- | --- | --- |
| Urumqi | 1 | Yanhu | A(10) |
| Turpan Basin | 2 | Tuokexun | A(10) |
| Tarim Basin | 3 | Kumush | A(11) |
|  | 4 | Heshuo | A(11) |
|  | 5 | Wushitala | A(11) |
|  | 6 | Tashidian | A(10);B(1);C(1) |
|  | 7 | Luntai | A(11) |
|  | 8 | Kuqa | A(10) |
|  | 9 | Yanshuigou | A(10) |
|  | 10 | Tiereke | A(10) |
|  | 11 | Chaerqi | A(10) |
|  | 12 | Keping | A(12) |
|  | 13 | Patrul | D(10) |
|  | 14 | Atushi | D(10) |
|  | 15 | Mayikake | D(10) |
|  | 16 | Wuheshalu | D(8);A(2) |
|  | 17 | Pishan | E(10) |
|  | 18 | Cele | E(10) |
|  | 19 | Qiemo1 | E(3);F(7) |
|  | 20 | Qiemo2 | E(4);F(6) |
|  | 21 | Ruoqiang | A(2);E(4);F(4) |
| Junggar Basin | 22 | Qitai1 | G(11) |
|  | 23 | Beitashan | A(2);G(8) |
|  | 24 | Qitai2 | A(1);G(9) |
|  | 25 | Karamay | A(2);G(4);H(1);I(3) |
|  | 26 | Wuchang | A(2);I(9) |
|  | 27 | Alashankou | G(11) |
|  | 28 | Hefeng | G(10) |
|  | 29 | Burqin | G(9);J(1) |
| Hami Basin | 30 | Hami | A(10) |
| Hexi Corridor | 31 | Liuyuan | A(9);K(2) |
|  | 32 | Guazhou | A(6);L(1);M(4) |
|  | 33 | Jinta | A(2);M(8) |
| AlxaDesert | 34 | Ejina1 | A(3);M(7) |
|  | 35 | Ejina2 | A(2);M(8) |
| Hexi Corridor | 36 | Yumen | M(10) |
|  | 37 | Akesai | M(10) |
|  | 38 | Subei | M(10);N(1) |
| Qaidam Basin | 39 | Lvcaoshan | M(10) |
|  | 40 | Mahuanggou1 | M(9);O(2) |
|  | 41 | Mahuanggou2 | M(8);O(2) |
|  | 42 | Daqaidam | M(12) |
|  | 43 | Golmud | M(10) |
|  | 44 | Ruomuhong | M(12) |
| Hexi Corridor | 45 | Xuebai | P(10) |

**TABLE S3** | Spatial autocorrelation tests of ensemble predictions under three pseudo-absence replicates.

| Model | Weight | Moran.I | SD | p.value | Sig | p.adj | Sig.adj |
| --- | --- | --- | --- | --- | --- | --- | --- |
| EMmean | PA1 | 0.0261 | 0.0056 | p<0.001 | *** | p<0.001 | *** |
| EMmean | PA2 | 0.0170 | 0.0055 | p<0.001 | *** | p<0.001 | *** |
| EMmean | PA3 | 0.0201 | 0.0053 | p<0.001 | *** | p<0.001 | *** |
| EMmedian | PA1 | 0.0252 | 0.0056 | p<0.001 | *** | p<0.001 | *** |
| EMmedian | PA2 | 0.0134 | 0.0055 | p<0.01 | ** | p<0.01 | ** |
| EMmedian | PA3 | 0.0197 | 0.0053 | p<0.001 | *** | p<0.001 | *** |
| EMca | PA1 | 0.0244 | 0.0056 | p<0.001 | *** | p<0.001 | *** |
| EMca | PA2 | 0.0120 | 0.0055 | p<0.05 | * | p<0.005 | * |
| EMca | PA3 | 0.0168 | 0.0053 | p<0.001 | *** | p<0.001 | *** |
| EMwmean | PA1 | 0.0261 | 0.0056 | p<0.001 | *** | p<0.001 | *** |
| EMwmean | PA2 | 0.0170 | 0.0055 | p<0.001 | *** | p<0.001 | *** |
| EMwmean | PA3 | 0.0201 | 0.0053 | p<0.001 | *** | p<0.001 | *** |
| CEM | PA1 | -0.0033 | 0.0052 | P>0.05 | ns | P>0.05 | ns |
| CEM | PA2 | -0.0035 | 0.0051 | P>0.05 | ns | P>0.05 | ns |
| CEM | PA3 | -0.0040 | 0.0049 | P>0.05 | ns | P>0.05 | ns |

Consensus Ensemble Model (CEM): A grid cell was classified as “species present” only if all four ensemble modeling methods (committee averaging [EMca], mean [EMmean], median [EMmedian], and weighted mean [EMwmean]) predicted presence. PA1–PA3 represent three independent pseudo-absence replicates.
